# Thermal energy storage and thermal conductivity properties of Octadecanol-MWCNT composite PCMs as promising organic heat storage materials

**DOI:** 10.1038/s41598-020-64149-3

**Published:** 2020-06-08

**Authors:** Amir Al-Ahmed, Ahmet Sarı, Mohammad Abu Jafar Mazumder, Gökhan Hekimoğlu, Fahad A. Al-Sulaiman, Inamuddin

**Affiliations:** 1 Center of Research Excellence in Renewable Energy (CORERE), King Fahd University of Petroleum & Minerals, Dhahran, 4000 Saudi Arabia; 2 Karadeniz Technical University, Department of Metallurgical and Material Engineering, 61080 Trabzon, Turkey; 3 Department of Chemistry, King Fahd University of Petroleum & Minerals, Dhahran, Saudi Arabia; 4 Advanced Functional Materials Laboratory, Department of Applied Chemistry, Faculty of Engineering and Technology, Aligarh Muslim University, Aligarh, 202 002 India; 5 Chemistry Department, Faculty of Science, King Abdulaziz University, Jeddah, 21589 Saudi Arabia

**Keywords:** Chemistry, Energy

## Abstract

Fatty alcohols have been identified as promising organic phase change materials (PCMs) for thermal energy storage, because of their suitable temperature range, nontoxicity and can be obtained from both natural and synthetic sources. Like all other organic PCMs, octadecanol (OD) as PCM suffers from low thermal conductivity (TC). In this work, to enhance its TC, it was grafted on the functionalized MWCNT and were used as a conductive filler to enhance overall thermal properties of OD in a composite PCM (CPCMs) structure. The OD/OD-g-MWCNT sample showed better dispersion within the composites and the presence of additional OD boosted the overall heat storage enthalpy compared to that of plane composite sample with OD/MWCNT. In a non-quantitative approach, it was observed that, any increase in grafting ratio of OD increases the heat storage enthalpy of the composites. The heat storage enthalpy of (267.7 J/g) OD/OD-*g*-MWCNT(4:1)-5wt% composite PCM had reached very close to the heat storage enthalpy value of pure OD (269.3 J/g), and much higher than that of OD/MWCNT-5wt% (234.5 J/g). Champion sample i.e. OD/OD-*g*-MWCNT (4:1)-5wt%, showed good heat storage enthalpy, cycling performance, thermal stability and TC enhancement by 262.5%.

## Introduction

Thermal energy is one of the major sources of natural green energy. Various methods have been developed to utilize this energy efficiently into our energy mix. Among these methods, phase change material (PCM) based latent heat storage has gained considerable attention, mainly, due to its simplicity, higher efficiency, and wide ranges of PCMs^1,2^. Among the different PCMs, inorganic PCMs have been heavily studied, due to their good thermal conductivity (TC) and heat of fusion. However, these materials suffers from supercooling, phase separation behavior, corrosive to metals, and also has toxicity^3,4^. On the other hand, organic PCMs showed better prospects in several thermal energy storage (TES) applications, mainly due to their good storage capacity, nontoxicity, and environmental safety^5,6^. These materials based heat storage technologies are getting applications in smart and green buildings, electronic devices, and so on^7–9^.

Among the different organic PCMs, fatty alcohols have received scant attention, though these alcohols can be obtained from both natural and petroleum sources; they are nontoxic and have suitable melting temperate for low temperature heat storage. Only a handful of reports exist where, fatty alcohols are studied for their TES properties^10,11^. Like other organic PCMs, they also suffer from low TC and leakage issues. So various materials, such as carbon-based materials, metal foams etc., were incorporated to improve the TC. Huang *et al*.^12^, prepared myristyl alcohol based composite PCMs (CPCMs) with nickel and copper foams by vacuum melting infiltration method. The heat storage enthalpy of CPCMs was decreased by 3–29%, while their TC was improved by 1.80-7.51 times compared with that of pure myristyl alcohol. In another work, they^13^ added carbon fiber to cetyl alcohol/high-density polyethylene composite to enhance its TC. TC value of the shape-stabilized CPCM was nearly 1.25 and 1.22 times higher than the CPCM without carbon fiber, respectively in liquid and solid state. In another similar work, Tang *et al*.^14^ prepared form-stable CPCM with stearyl alcohol and high density polyethylene. Here, they used expanded graphite as a TC enhancer. This CPCM showed a constant melting temperature of 57 °C with heat storage enthalpy of 200 J/g and enhanced TC by 240% with addition of just 3% expanded graphite. Wang *et al*.^15^ prepared 1-octadecanol (OD)/graphene oxide (1.5 wt%) CPCM by self-assembly method and improved its TC value by 1.5 times compared to that of pure OD. In another study, Tang *et al*.^16^ adsorbed OD in hierarchical porous polymer and prepared shape-stable CPCM. They were able to achieve nearly 75% of adsorption and observed 29.8% drop of the heat storage enthalpy value than that of the pure OD.

It is still challenging to minimize the loss of the overall heat storage enthalpy capacity, when a conducting filler with higher TC is introduced to improve the thermal properties of the organic PCMs^17,18^. In this regard, several attempts have been made to enhance TC without adversely affecting the heat storage enthalpy of PCMs. Mu and Li^19^ grafted lauric acid with graphene aerogel by an esterification reaction. The TC value of this composite was increased by 352.1% and 32.6% compared with that of pure lauric acid and lauric acid/graphene aerogel, respectively. The melting enthalpy and freezing enthalpy of the composite was found to be 207.3 J/g and 205.8 J/g, respectively. Xiao *et al*.^20^ dispersed carbon nanotubes (CNT), oxidized-CNT and grafted-CNT into palmitic acid at a mass ratio of 1:100. The latent heat of palmitic acid/grafted-CNT even exceeded that of pure PA and also showed higher TC. Tu *et al*.^21^ grafted polyethylene glycol on graphene oxide and introduced into the polyethylene glycol to achieve enhanced graphene oxide dispersion and heightened the mobility of the PEG chains. The heat storage enthalpy and thermal conductivity were enhanced compared to that of the composite with only graphene oxide. Li *et al*.^22^ grafted three different polyalcohols octanol, tetradecyl alcohol and stearyl alcohol on the functionalized CNT and mixed with paraffin to prepare the CPCMs. They observed polyalcohols with higher number of carbon gave better TC but lower heat storage enthalpy. However, the grafted PCMs showed better dispersion than that of pure CNT within paraffin due to reduction of the length of the CNT after the grafting and improved compatibility.

In most of the previous reports, it was observed that the direct mixing of carbon-based nano fillers within fatty acids and fatty alcohols was resulted an ununiformed dispersion, which eventually affected thermal property. There are very few attempts, where some organic PCMs were grafted on the carbon-based nano fillers, but still they did not study the effect of the grafting amount, besides; investigate the cycling TES reliability of the resulting CPCM. Even in all these cases, the modified conductive filler was incorporated in to the other kind of PCMs than that of the grafted one. Therefore, to have better compatibility, to achieve better dispersion in the both solid and liquid state, to improve thermal properties of OD, it was grafted on the functionalized multi-walled carbon nanotube (MWCNT) in three different grafting ratios (1:1, 2:1 and 4:1) by a two-step chemical treatment process. The grafted and non-grafted MWCNT were subsequently used as conductive fillers with pure OD in 5 wt% to achieve novel kinds of CPCMs. This supposed to give a homogeneous dispersion of the prepared conductive filler within the CPCM in both liquid and solid state. It was also expected to achieve a better TC, good heat storage enthalpy, thermal stability and cycling TES properties. Thermal properties of the CPCM with grafted MWCNT (OD/OD-*g*-MWCNT) were also compared with that of CPCM with pure MWCNT (OD/MWCNT) and even with fatty alcohol included-CPCMs previously reported in literature. The produced CPCM samples showed superior thermal properties as well as reliable cycling TES properties.

## Experimental

### Materials

1-Octadecanol (OD; purity degree 95%) selected as organic PCM was procured from Sigma-Aldrich. Thionly chloride and nitric acid (HNO_3_) were purchased from BDH Chemicals Ltd, and used as received. MWCNT was purchased from cheap tube USA, and used without any modification.

### Synthesis of OD-*g*-MWCNT pre-composites

*Carboxylation of MWCNT*. In a typical process described elsewhere^23^, MWCNT (5 g) and 350 mL of HNO_3_ solution (60% v/v) were mixed in a 500 mL round bottom (RB) flask and sonicated for 3 h. The flask content was refluxed at 120 °C for 12 h and then cooled at room temperature. The product was vacuum filtrated using microporous membrane with pore size of 0.22 µm. The filtrate was washed thoroughly with de-ionized water until neutral pH was achieved. Finally, the functionalized MWCNT were dried in vacuum oven to a constant weight at 60 °C.

#### Conversion of MWCNT-COOH to MWCNT-COCl

Carboxyl functionalized MWCNT (4 g) and thionly chloride (60 mL) were mixed slowly at room temperature in a 250 mL- RB flask. Then, the reaction mixture was refluxed at 80 °C for 24 h. Finally, thionly chloride was distilled off using a liquid nitrogen trap, and the product was washed with de-ionized water to obtain acyl chloride-functionalized MWCNT (MWCNT-COCl).

#### OD-grafted MWCNT (OD-g-MWCNT) pre-composite

**Figure 1:**
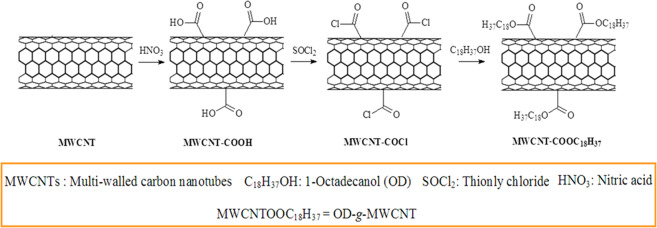
The reaction route used in the synthesis of functionalized MWCNT and 1-Octadecanol grafted MWCNT (OD-*g*-MWCNT) pre-composites.

At first, OD was melted on a temperature-controlled heater at 65 °C and combined with MWCNT-COCl at weight ratio of 1:1. This mixture was refluxed at 75 °C for 48 h while stirring. After cooling to room temperature, the product was washed with de-ionized water to remove any unreacted OD. Finally, obtained OD-*g*-MWCNT(1:1) pre-composite was dried in vacuum oven at 60 °C for 12 h. Two other pre-composites were synthesized at 2:1 and 4:1 weight ratios of OD and MWCNT-COCl under the same reaction conditions described as above. The reaction route used in the synthesis of functionalized MWCNT and OD-*g*-MWCNT pre-composites is illustrated in Fig. 1.

**Figure 2:**
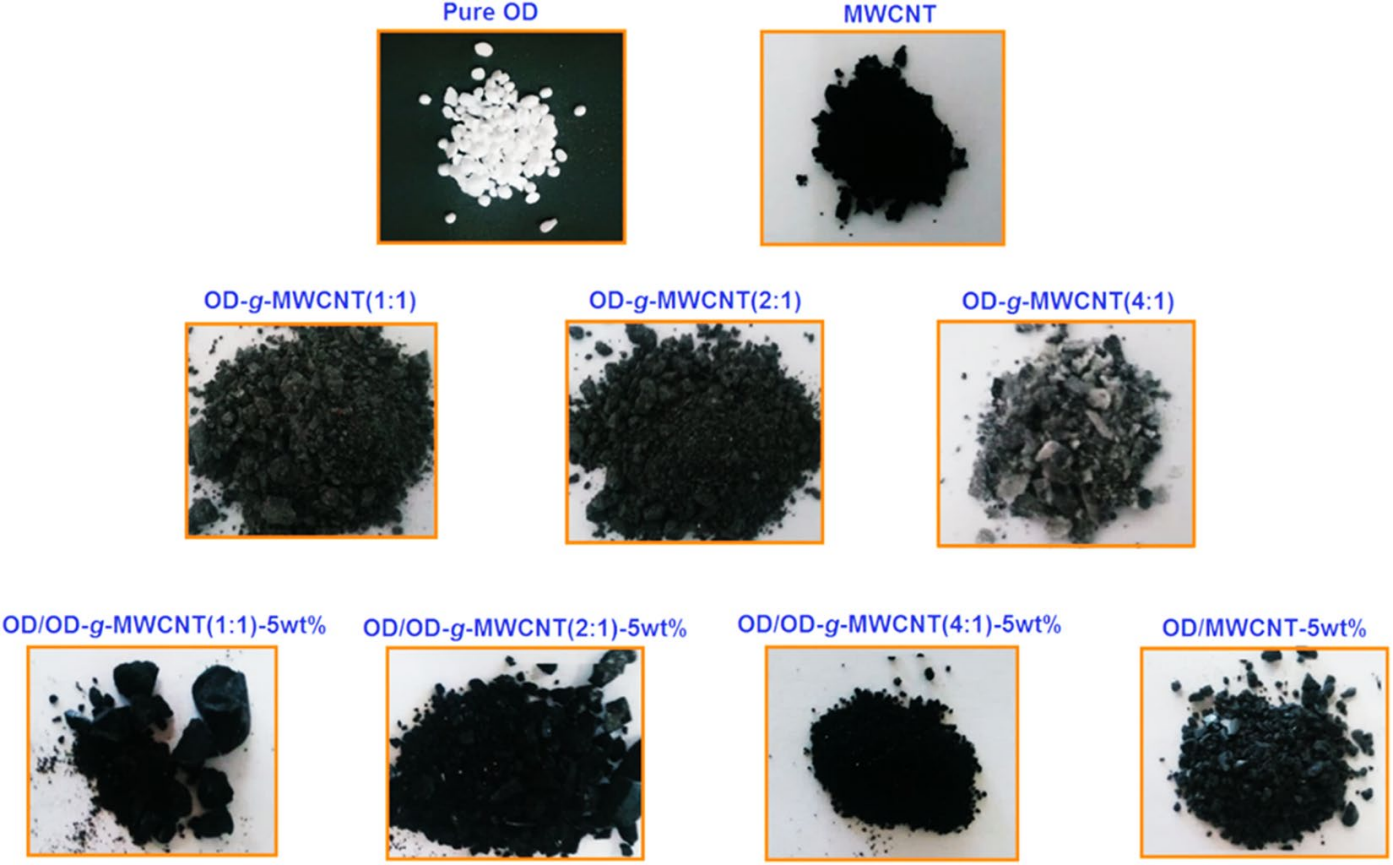
Photograph images of OD, MWCNT, the synthesized OD-*g*-MWCNT pre-composites and prepared CPCMs, OD/OD-*g*-MWCNT-5wt% and OD/MWCNT-5wt%.

Here, OD was melted on a temperature-controlled heater at 65 °C and mixed with OD-*g*-MWCNT (1:1) in percentage mass ratio of 95/5. The obtained sol-gel compositions were mixed by a temperature-controlled shaker at 200 rpm for 30 min to get homogenous dispersions. The composites were cooled at room temperature and then grounded for characterization and further use. Same procedure was used for the preparation of other composites with 5 wt% OD-*g*-MWCNT pre-composite with grafting ratio of 2:1, 4:1 and pure MWCNT. The final CPCMs containing 5 wt% grafted and non-grafted MWCTs were sealed as OD/OD-*g*-MWCNT(1:1)-5wt%, OD/OD-*g*-MWCNT(2:1)-5wt%, OD/OD-*g*-MWCNT(4:1)-5wt% and OD/MWCNT-5wt%. The photograph images of OD, MWCNT, the synthesized OD-*g*-MWCNT pre-composites and prepared CPCMs, OD/OD-*g-*MWCNT-5wt% and OD/MWCNT-5wt% are shown in Fig. 2.

### Dispersion stability experiment

The pre-weighed amount of OD was melted at 65 °C in glass tubes. OD-*g-*MWCNT and MWCNT pre-composites were added to the melted OD at 5 wt% of the total weight of the composite samples. The composite samples, OD/OD-*g-*MWCNT-5wt% and OD/MWCNT-5wt%, were mixed using a mini centrifuge at 100 rpm for 15 min to obtain homogenous distribution. Then, in order to observe the dispersion stability, both composite samples were maintained at 65 °C for 1 h without stirring.

### Characterization

Grafting of OD with the functionalized MWCNT was studied by FTIR spectroscopy (Perkin Elmer, 16 F PC FTIR) technique. It was also used to investigate the chemical structures of pure OD, MWCNT, OD-*g*-MWCNT pre-composites, OD/OD-*g*-MWCNT-5wt% and OD/MWCNT-5wt% as CPCMs. The spectra were taken in the wavelength range of 4000-400 cm^−1^. The surface morphologies of the CPCMs were studied by scanning electron microscopy (SEM) (LEO 440 model). The samples were coated with gold to provide electrical conductivity. The crystalline structures of the CPCMs were studied using a XRD instrument (PANalytical X’-Pert3 model) with Cu (Kα = 1.5406 Å) irradiation at a step size of 0.0131°.

The phase change temperatures and heat storage enthalpy of pure OD, prepared pre-composites and CPCMs were measured using a differential scanning calorimetry (DSC) (Hitachi-DSC 7020 model) at a heating/cooling rate of 3 °C/min. This measurement was repeated three times and the mean deviation in phase change temperatures and heat storage enthalpy values were determined (minor deviation of ± 0.12 °C and minor error of ±1.03% were determined). Thermal degradation temperatures of OD, pre-composites and CPCMs were determined at heating ramp of 10 °C/min by thermal gravimetric analysis (TGA) (Perkin-Elmer model) at 30-600 °C. The temperature measurement for each sample was also taken three times and the mean deviation was determined as ±1.5 °C.

The TCs of pure OD, pre-composites and CPCMs were measured using a thermal properties analyzer (Decagon KD2 pro Devices, Inc., USA). The measurements were conducted at 20 ± 1 °C by using KS1. sensor with the maximum discrepancy of ±10% W/m K. To minimize inaccuracy, the measurements were repeated three times at 10 minutes intervals.

To investigate the effect of thermal cycling on the chemical structures, crystalline phases and TES properties of the synthesized CPCMs, a thermal cycling test was carried out using a thermal cycler (BIOER TC-25/H model). Throughout the test, the samples were melted and solidified repeatedly until targeted cycling number of 500 was achieved. After the cycling test, FTIR, XRD and DSC analyses of these CPCMs were performed again to compare results obtained for the fresh CPCM samples.

## Results and Discussions

### Chemical structure characterization

**Figure 3:**
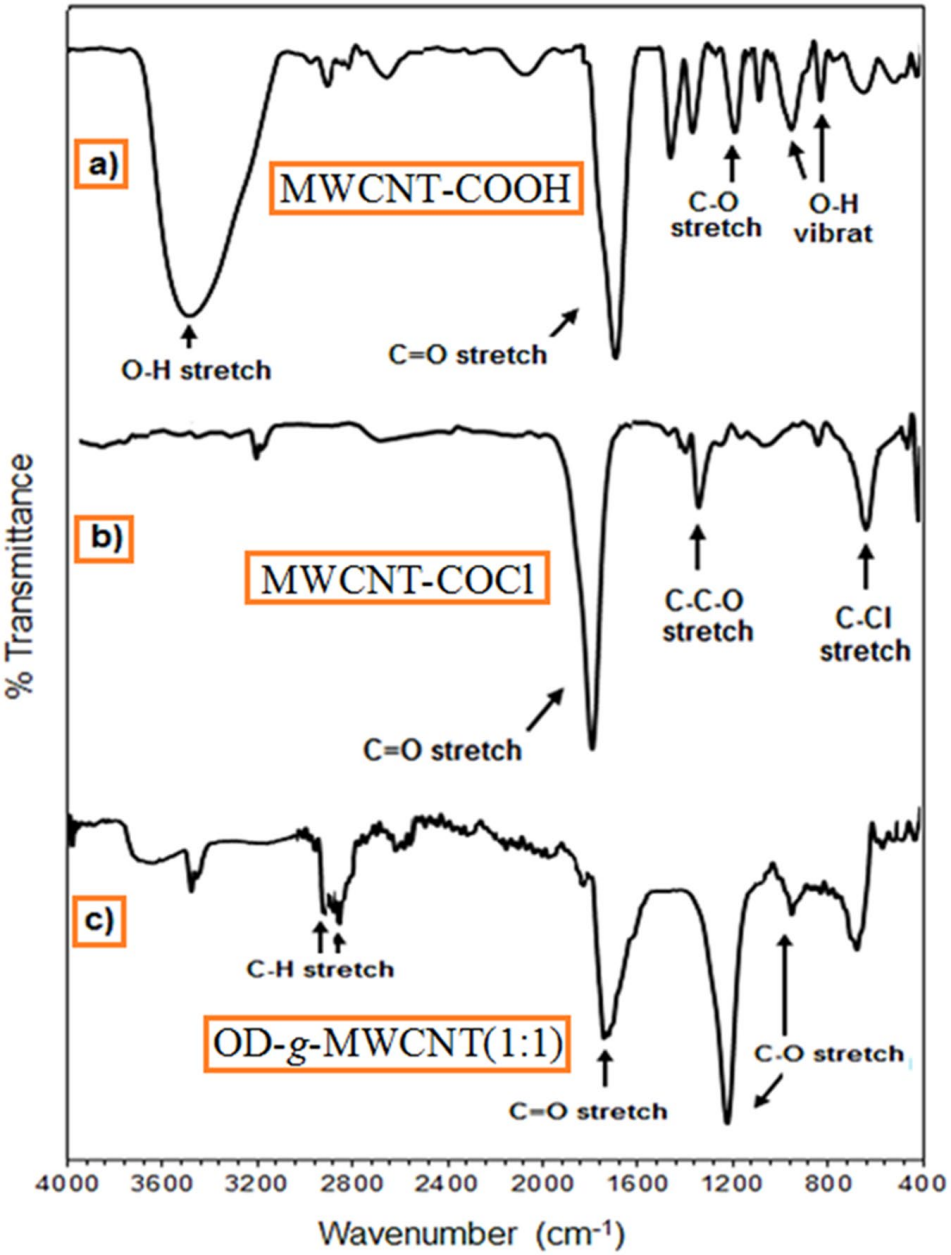
FTIR spectra of (**a**) MWCNT-COOH, (**b**) MWCNT-COCl and (**c**) OD-*g*-MWCNT(1:1).

The FTIR spectra of the synthesized MWCNT-COOH, MWCNT-COCl and OD-*g*-MWCNT (1:1) are given in Fig. 3. In the spectrum of MWCNT-COOH, the broad peak in between at 3250 and 3650 cm^−1^ confirms the presence of hydroxyl (–OH) groups present in MWCNT^22^. The absorption peak at 1690 cm^−1^ represents the stretching of C=O bond of –COOH group as the symmetric and asymmetric stretching vibrations of COOH were appeared at 1471 and 1359 cm^−1^, respectively^24^. In addition, the peaks appeared at 958 and 829 cm^−1^ are assigned to vibration of –OH groups^25^. In the spectrum of MWCNT-COCl, the absorption peak at 1776 cm^−1^ is related with the stretching vibration of C=O of acid chloride. The absorption peaks at 1328 and 639 cm^−1^ confirmed the presence of C–O and C–Cl stretching of acid chloride functionalized MWCNT. The peak at 1736 cm^−1^ is attributed to the stretching of C=O, which is from OD-*g*-MWCNT. On the other hand, in the spectrum of OD-*g*-MWCNT pre-composite, the intensity of –OH absorption peak between at 3250 and 3650 cm^−1^ was significantly decreased after grafting. However, a small peak seen in this range is associated with the vibration of –OH groups of OD, which were not grafted with MWCNT-COCl. Moreover, the peaks detected at 2924 and 2836 cm^−1^ are identified with the stretching vibration of -CH_2_ and -CH_3_ groups that come from OD. All these confirms that the OD was successfully grafted on the MWCNT^22^.

**Figure 4:**
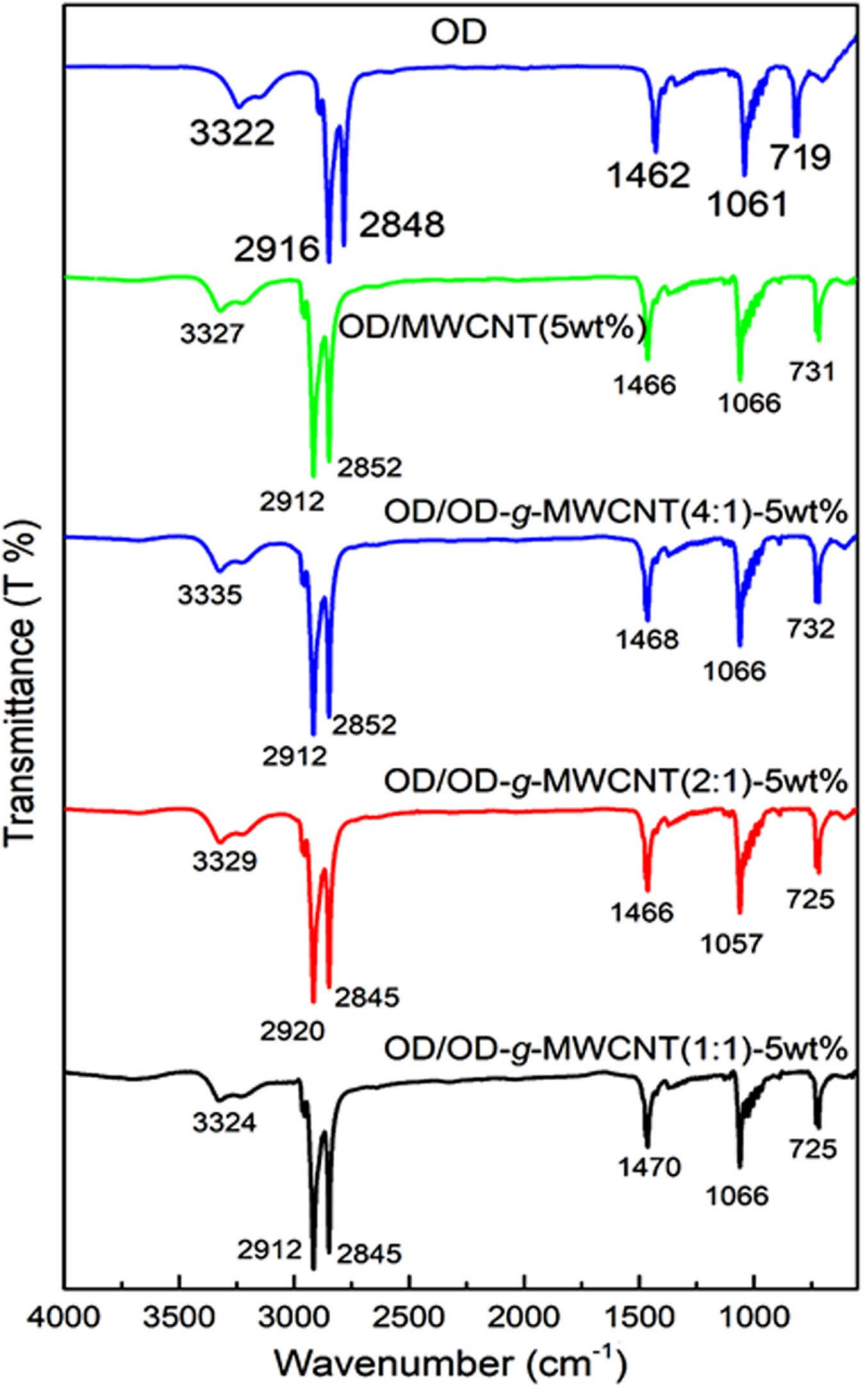
FTIR spectral results of pure OD and the prepared CPCMs.

The FTIR spectra of pure OD, OD/OD-*g*-MWCNT(1:1)-5wt%, OD/OD-*g*-MWCNT(2:1)-5wt%, OD/OD-*g*-MWCNT(4:1)-5wt% and OD/MWCNT-5wt% composites are presented in Fig. 4. It is clear from the spectra that there is no additional peak in the spectrum of the CPCMs after mixing of OD with the grafted or non-grafted MWCNT. Slight shifts in the wavenumbers of some absorption peaks were observed for the CPCMs compared to that of pure OD, which can be due to physical interactions or could be identified as capillary and surface tension forces among them^11,22,23^.

**Figure 5:**
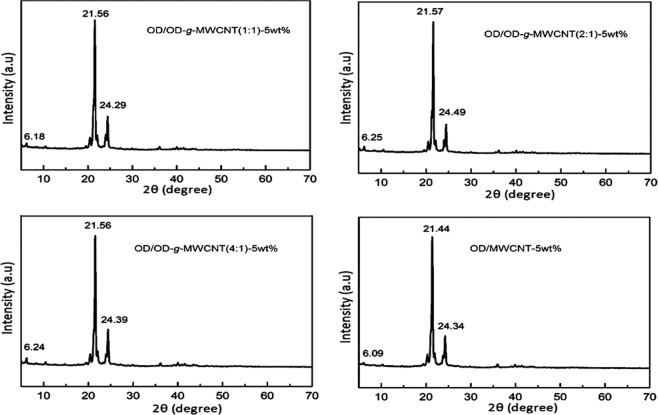
XRD results of the prepared CPCMs.

On the other hand, to investigate the effect of the grafted or non-grafted MWCNT on the crystal structure of the OD, the XRD analysis was conducted. Figure 5 shows the XRD analysis results obtained for OD/OD-*g*-MWCNT(1:1)-5wt%, OD/OD-*g*-MWCNT(2:1)-5wt%, OD/OD-*g*-MWCNT(4:1)-5wt% and OD/MWCNT-5wt% composites. All the typical diffraction peaks of OD is visible around 6°, 20°, 21.5°, 22°, and 24.5°, the doublet around 24.5° may be due the MWCNT^15^. Since the amount of the nanotubes is quite low, it is difficult to get the separate distinguishable peak of them^11,17,22^.

### Surface morphology and dispersion stability

**Figure 6:**
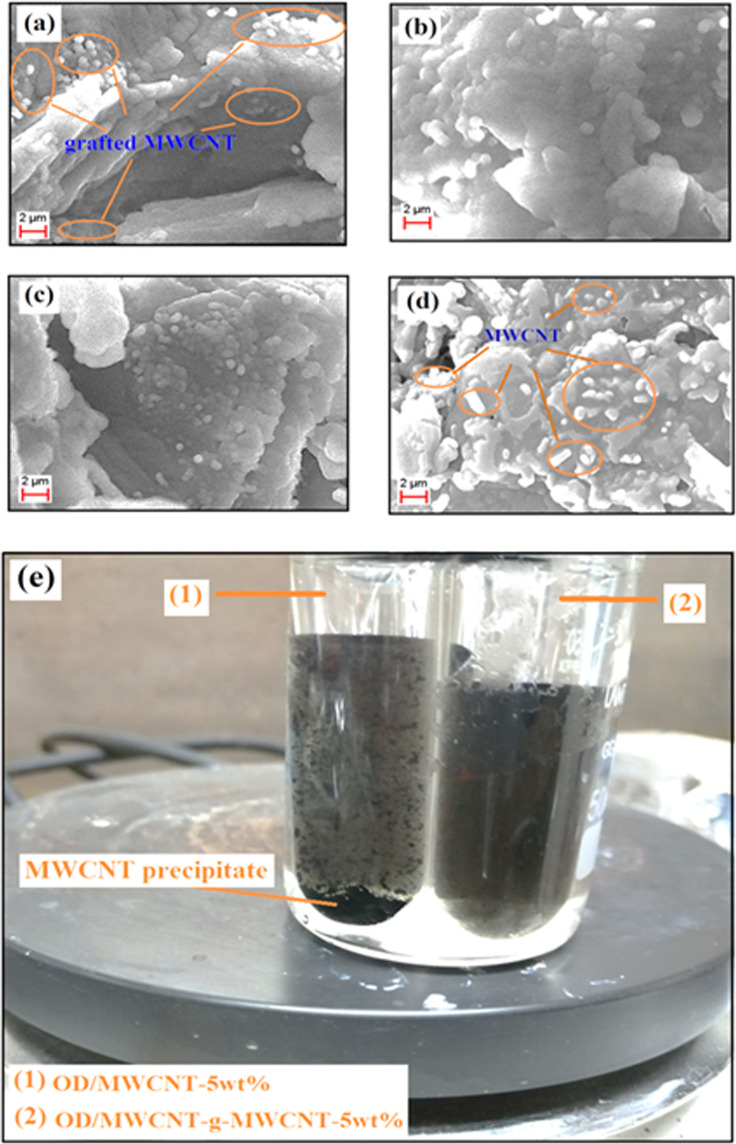
SEM photographs of (**a**) OD/OD-g-MWCNT(1:1)-5wt%, (**b**) OD/OD-g-MWCNT(2:1)-5wt%, (**c**) OD/OD-g-MWCNT(4:1)-5wt% (**d**) OD/MWCNT-5wt% (**e**) Photograph image of OD/OD-*g*-MWCNT-5wt% and OD/MWCNT-5wt% suspensions after 1 h-standby at 65 °C.

Surface morphology and distribution of the MWCNT within the CPCMs were investigated by SEM analysis. The obtained SEM images are presented in Fig. 6(a). As seen from the images of the OD/OD-*g*-MWCNT composites, the phase intensity of the grafted MWCNT on the surface of OD was decreased with decreasing its grafting ratio. The nanotubes were dispersed uniformly throughout the surface of OD and no agglomeration was observed. The uniformity of MWCNT in grafted or mixed state creates heat transfer network, which could provide effective heat conduction paths throughout the CPCM^21,22^.

Figure 6(b) shows the images of the dispersion stability of OD/OD-*g*-MWCNT-5wt% and OD/MWCNT-5wt% composite samples at 65 °C after 1 h-standby period. As clearly seen, some parts of MWCNT in the OD/MWCNT-5wt% sample began to precipitate while the sample OD/OD-*g*-MWCNT-5wt% maintained its stable suspension. These results indicate that grafted MWCNT created more stable suspension with melted OD compared to the pure MWCNT. It is probably a result of relatively stronger colloidal attractions between OD-*g*-MWCNT and OD. The similar observation was reported for the acid modified carbon nanotubes/erythritol composite suspension^26^.

### TES properties of the synthesized pre-composites and CPCMs

**Figure 7:**
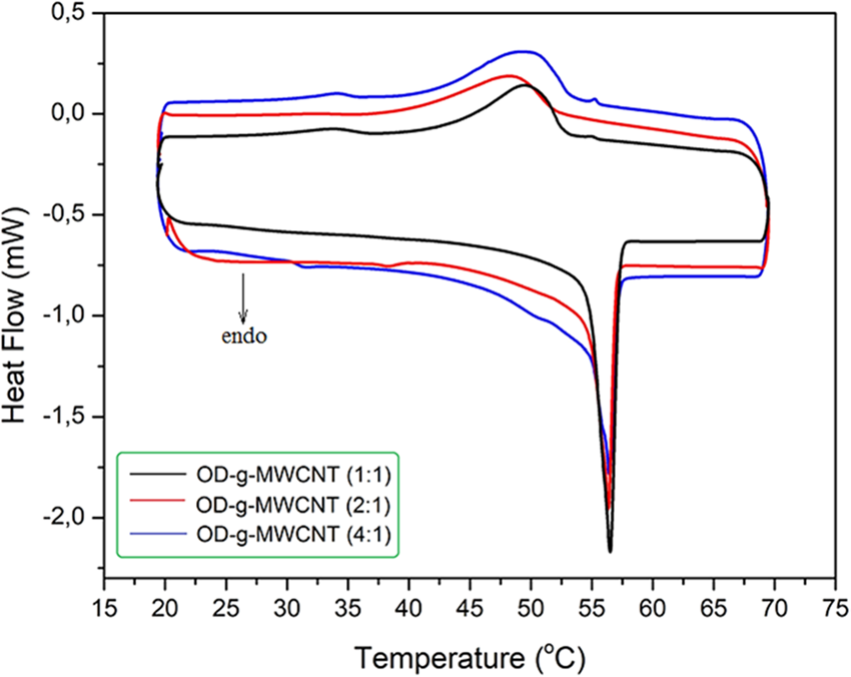
DSC thermograms of OD-*g*-MWCNT products synthesized as pre-CPCMs.

The DSC results of the synthesized OD-*g*-MWCNT pre-composite samples are presented in Fig. 7. It was observed that, the phase change temperatures of OD-*g*-MWCNT (1:1), OD-*g*-MWCNT (2:1) and OD-*g*-MWCNT (4:1) somewhat decreased by 1.1, 1.0 and 0.9 °C for melting phase change and 2.4, 0.6 and 1.3 °C for freezing phase change in comparison with that of pure OD. These irregularly shifts could be resulted because of its conversion to new chemical structures. Both heat storage capacities measured for melting and freezing of all grafted samples are quite low, due to very small amount of OD present in the grafted samples. However, both heat storage capacities increased with the increasing amount of OD in the grafted samples.

**Table 1:** The TES properties of pure OD, synthesized pre-composites and CPCMs.

PCM or composite PCM	Melting temperature (°C)	Freezing temperature (°C)	Melting enthalpy (J/g)	Freezing enthalpy (J/g)
OD	55.5 ± 0.12	54.7 ± 0.13	269.3 ± 2.7	−268.2 ± 2.6
OD/OD-g-MWCNT(1:1)-5wt%	55.3 ± 0.13	55.4 ± 0.11	241.2 ± 2.4	−238.4 ± 2.4
OD/OD-g-MWCNT(2:1)-5wt%	55.0 ± 0.12	55.4 ± 0.10	249.3 ± 2.5	−244.2 ± 2.5
OD/OD-g-MWCNT(4:1)-5wt%	54.6 ± 0.11	55.6 ± 0.13	267.7 ± 2.7	−263.6 ± 2.6
OD/MWCNT-5wt%	55.1 ± 0.12	55.8 ± 0.14	234.5 ± 2.3	−230.3 ± 2.2

**Figure 8:**
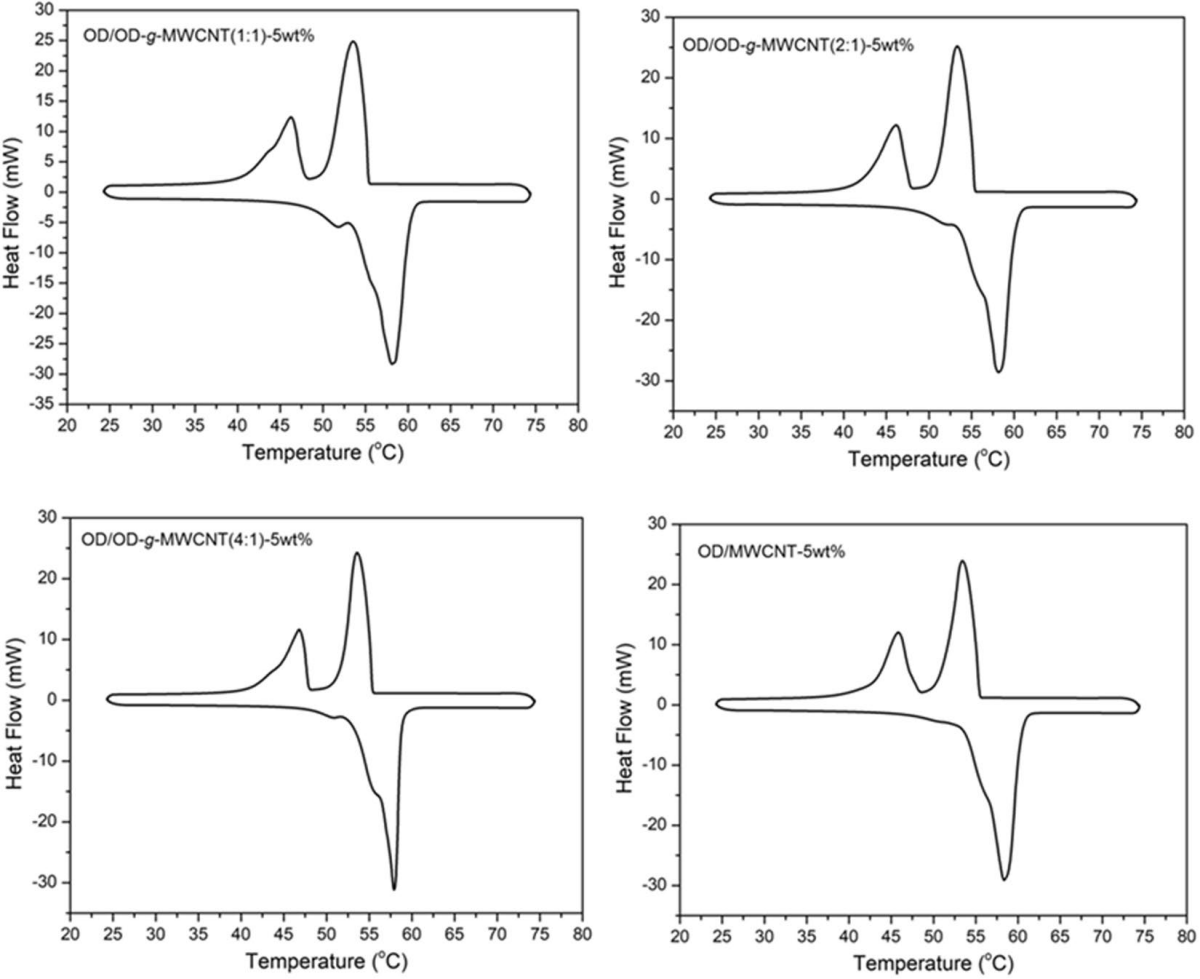
DSC thermograms of OD/OD-*g*-MWCNT and OD/MWCNT CPCMs.

The DSC thermograms of prepared OD/OD-*g*-MWCNT composites are presented in Fig. 8 and the TES data derived from these thermograms is given in Table 1. All of the CPCMs melts and solidifies in temperature range of 54.6-55.3 °C and 55.4-55.8 °C, respectively. When compared to these results with those of pure OD, it was found that the change in melting temperature of OD/OD-*g*-MWCNT (1:1)-5wt%, OD/OD-*g*-MWCNT (2:1)-5wt%, OD/OD-*g*-MWCNT (4:1)-5wt% and OD/MWCNT-5wt% composites were in the range of (-0.2) – (-0.9) °C and the change in freezing temperature of them was in the range of 0.7–1.1 °C. In addition, the heat storage capacities of the CPCMs increased in parallel with heat storage capacity of the corresponding pre-composite added to OD. In fact, the CPCM sample with OD-*g*-MWCNT (4:1) showed the highest heat storage capacity of 267.7 ± 2.7 and 263.03 ± 2.6 J/g, respectively for its melting and freezing period, which were very close to that of pure OD.

Additionally, when compared with the results obtained for the OD/MWCNT composite, its heat storage enthalpies were found to be lower than the other three CPCM samples. As it is visible from the tabulated data in Table 2, the CPCMs containing grafted and non-grafted MWCNT prepared in this work showed relatively higher heat storage capacities than those of previously reported similar PCMs^15,16,19,20,22^. It became clear that the grafting helped to achieve better distribution of MWCNT and the additional OD present in the grafted MWCNT also contributed to overall heat storage capacities of the PCMs.

**Table 2:** Comparison of TES properties of the prepared CPCMs with that of different kind of composites in literature.

CPCM	Melting/solidification temperature (°C)	Melting/solidification enthalpy	Reference
Cetyl alcohol/HDPE/CF(5 wt%)	48.55/41.51	171.0/96.4	^13^
Stearyl alcohol/HDPE/EG(3 wt%)	55.81/57.04	200.2/118.3	^14^
Octadecanol/Graphene (1.5%)	57.31/56.77	239.5/208.4	^15^
Lauric acid(LA)/LA-Graphene	43.51/40.27	207.2/208.5	^19^
Palmitic acid/CNTs	Not reported	187.1/Not reported	^20^
Palmitic acid/oxidized-CNTs	Not reported	194.0/Not reported	^20^
Palmitic acid/grafted CNTs	Not reported	201.7/Not reported	^20^
Polyethylene glycol(PEG)-g-Graphene oxide (GO)(5 wt%)	53.06 /43.56	166.1/159.6	^21^
PEG/GO(5 wt%)	54.91 /43.30	168.9/174.9	^21^
OD/MWCNT-5wt%	54.7/55.4	226.7/224.1	*This study*
OD/OD-*g*-MWCNT(4:1)-5wt%	55.4/55.3	267.7/263.6	*This study*

### Effect of thermal cycling on TES properties of the prepared CPCMs

**Figure 9:**
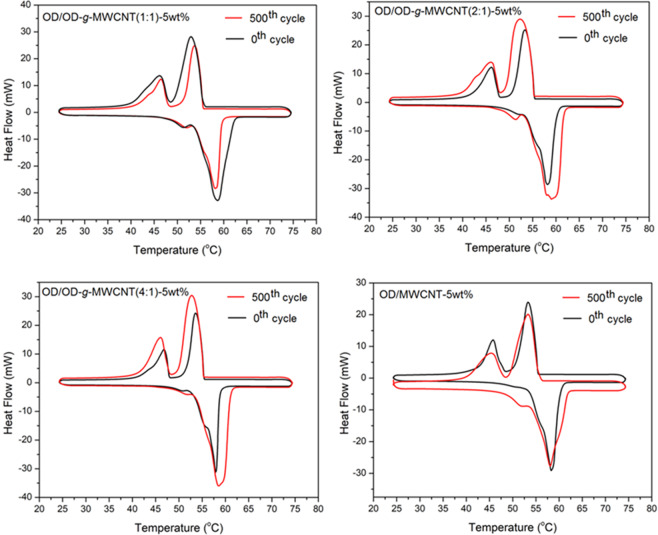
DSC thermograms of OD/OD-*g*-MWCNT and OD/MWCNT CPCMs before and after 500 cycles.

The cycling TES reliability of a PCM can be considered as its TES life after its long-term utilization^16,24,26^. With this respect, a consecutive thermal cycle test was adapted to all prepared composite PCMs and the DSC thermograms obtained after 500 cycles were indicated in Fig. 9. By comparing the data in Table 1 and Table 3, the change in melting temperature of OD/OD-*g*-MWCNT (1:1)-5wt%, OD/OD-*g*-MWCNT (2:1)-5wt%, OD/OD-*g*-MWCNT (4:1)-5wt% and OD/MWCNT-5wt% composites were determined as −0.7, 0.1, 0.8 and −0.4 °C and the variation in their freezing temperatures was established by −0.2, −0.1, −0.3 and −0.4 °C, respectively. Furthermore, the heat storage capacity were reduced by 8.6, 8.0, 7.3 and 7.8 J/g for melting of the CPCMs while the heat storage capacity was diminished by 7.1, 6.7, 8.4 and 6.1 J/g for freezing of respective composite. These minor alterations proves good TES cycling stability of the prepared composite PCMs.

**Table 3:** The TES properties of the prepared CPCMs after 500 thermal cycles.

CPCM	Melting temperature (°C)	Freezing temperature (°C)	Melting enthalpy (J/g)	Freezing enthalpy (J/g)
OD/OD-*g*-MWCNT(1:1)-5wt%	54.6 ± 0.10	55.2 ± 0.12	232.6 ± 2.3	−231.3 ± 2.3
OD/OD-*g*-MWCNT(2:1)-5wt%	55.1 ± 0.13	55.3 ± 0.13	241.3 ± 2.4	−237.5 ± 2.4
OD/OD-*g*-MWCNT(4:1)-5wt%	55.4 ± 0.12	55.3 ± 0.12	260.4 ± 2.6	−255.2 ± 2.6
OD/MWCNT-5wt%	54.7 ± 0.13	55.4 ± 0.11	226.7 ± 2.3	−224.1 ± 2.3

### Effect of thermal cycling on chemical structures of the prepared CPCMs

**Figure 10:**
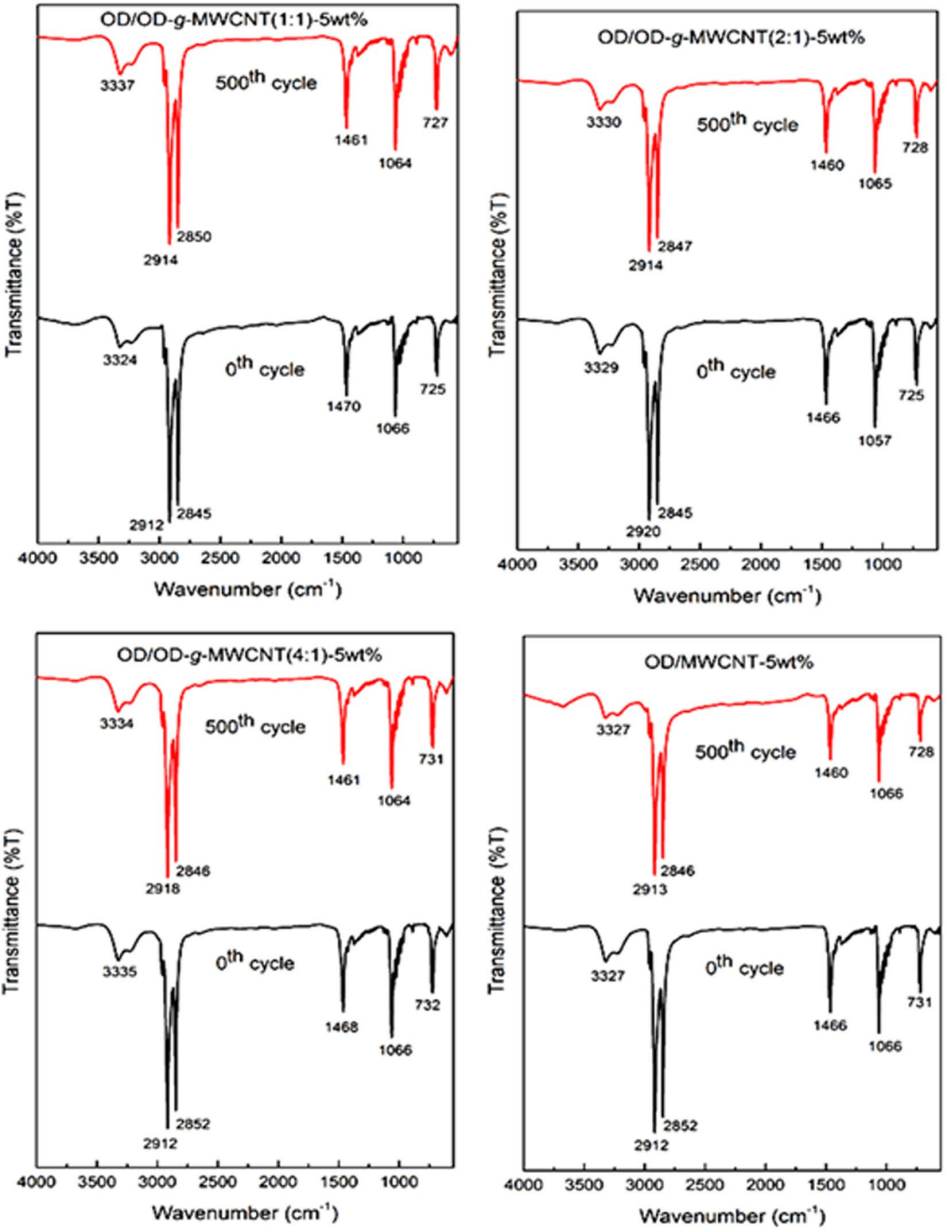
FTIR results of OD/OD-*g*-MWCNT and OD/MWCNT CPCMs before and after 500 cycles.

**Figure 11:**
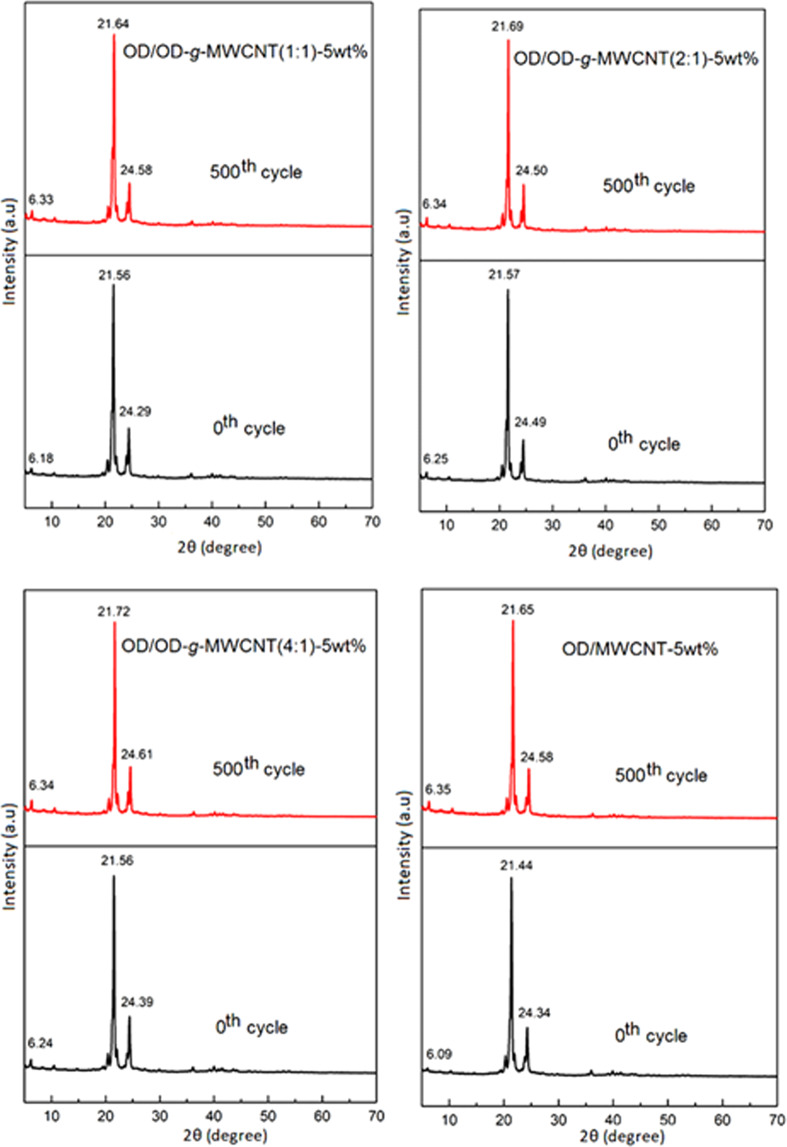
XRD results of OD/OD-*g*-MWCNT and OD/MWCNT CPCMs before and after 500 cycles.

The dependence of the chemical stability of PCM on long-term usage is one of the most imperative limits, which is worth considering during the selection stage. Accordingly, the FTIR and XRD analysis of four types of composite PCMs were repeated after 500 cycles. The results of cycled and non-cycled composite PCMs were compared in Figs. 10 and 11. As evident found from the FTIR spectrums, the profile and absorption wavenumbers of the main band peaks were nearly unchanged after the cycling process. In XRD, similar response was observed, without any new peaks. The 2*θ* ° values of the characteristic peaks were almost same after the cycling period. Both results confirms that all of the composite PCMs had unique cycling chemical stability.

### Thermal degradation behavior of the prepared composite PCMs

**Figure 12:**
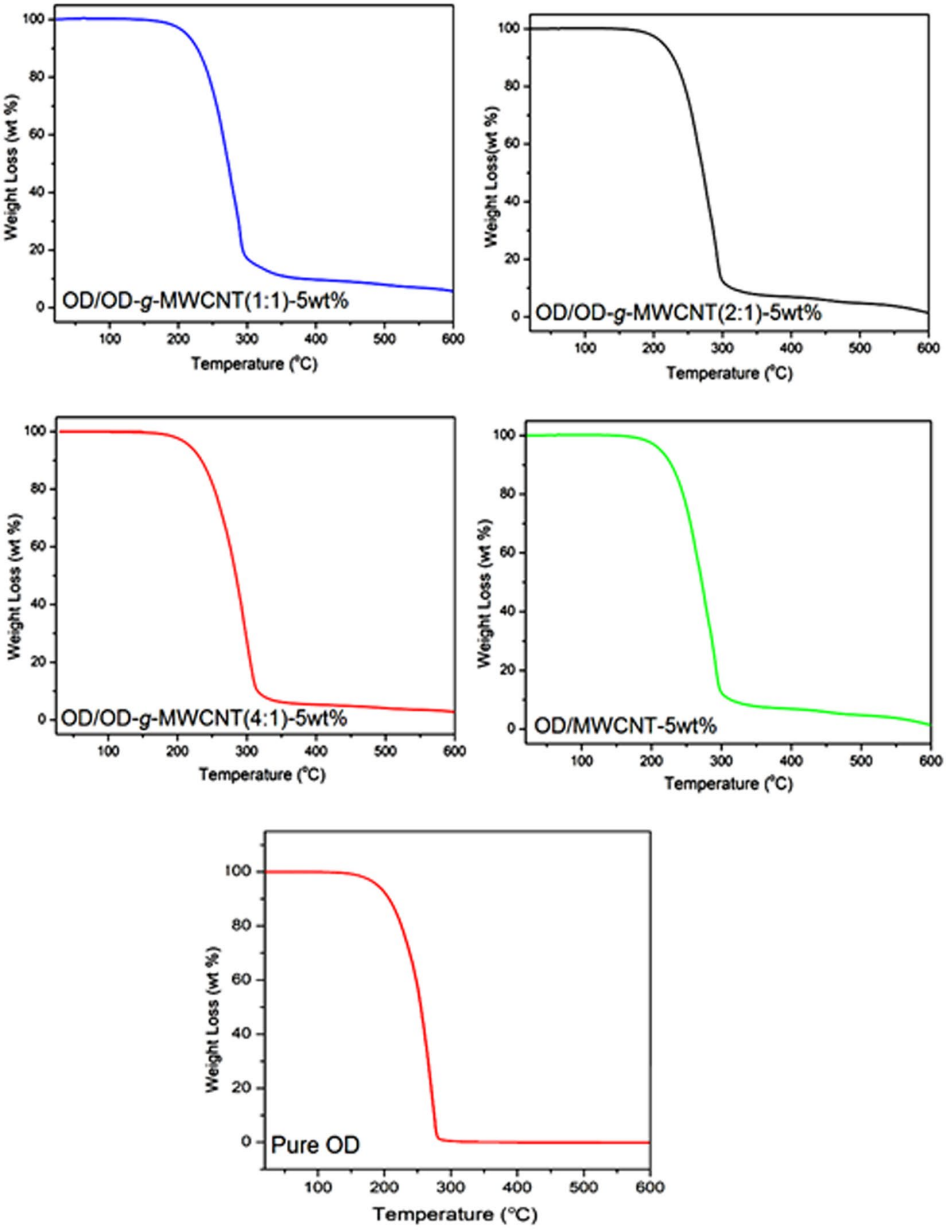
TGA curves of pure OD, OD/OD-*g*-MWCNT and OD/MWCNT CPCMs.

Thermal durability of pure OD and the fabricated composite PCMs were investigated by TGA analysis. As evident in Fig. 12, pure OD demonstrated a one-step thermal degradation between 190 °C and 295 °C. In similar form, the OD/MWCNT-5wt% composite PCM degraded thermally at only one stage corresponding to temperature range of 198-306 °C, which is slightly higher than the pure OD. OD grafted MWCNT i.e. OD/OD-*g*-MWCNT (1:1)-5wt%, OD/OD-*g*-MWCNT (2:1)-5wt%, OD/OD-*g*-MWCNT (4:1)-5wt% composite PCM samples also shown thermal decomposition with one-step gradation. The degradation started around 243 ± 1.3 °C, 235 ± 1.6 °C and 228 ± 1.4 °C and completed at 353 ± 1.7 °C, 337 ± 1.5 °C, and 318 °C ± 1.6 for the respective composite PCMs. A shift in initial and final degradation temperature was observed for all grafted samples. In the OD/OD-*g*-MWCNT (1:1)-5wt%, OD/OD-*g*-MWCNT (2:1)-5wt%, OD/OD-*g*-MWCNT (4:1)-5wt% composite PCM samples, the degradation temperature moved to higher values with the lower ratios of OD in the grafted samples. This manifested that the operating temperature and the thermal degradation temperature of pure OD was extended by the addition of grafted MWCNT. Similar results were also observed for different type of composite PCMs^27,28^.

### TC of the prepared pre-composites and composite PCMs

TC property is one of the important parameters considered during the PCM selection. PCMs with higher TC reduce the heat charging/discharging period during the operation of TES system. OD is an organic alcohol has low TC, which should be enhanced before utilization. One common approach is to add some nano particles with higher TC, like, graphite, CNT, graphene etc., to increase the TC property^29–31^. For this study, OD was grafted on the MWCNT and used as conductive filler. Pure OD has a TC value of 0.16 ± 0.01 W/m K. All other samples showed higher TC value than that of pure OD. The OD-*g*-MWCNT (1:1), OD-*g*-MWCNT (2:1), and OD-*g*-MWCNT (4:1) samples showed TC of 0.82 ± 0.04, 0.77 ± 0.05, and 0.69 ± 0.03 W/m K, respectively. TC value was measured as 0.57 ± 0.05, 0.76 ± 0.03, 0.61 ± 0.02, and 0.58 ± 0.04 W/m K, respectively for OD/MWCNT-5wt%, OD/OD-*g*-MWCNT (1:1)-5wt%, OD/OD-*g*-MWCNT (2:1)-5wt%, and OD/OD-*g*-MWCNT (4:1)-5wt% samples. The improvement in TC value in the grafted samples compared to that of pure OD is due to the presence of the MWCNTS, as expected the TC values were slowly reduces with the increasing amount of OD. The lower TC value of the OD/MWCNT-5wt% sample was mainly due to non-homogeneous distribution of MWCNTS and agglomeration as confirmed in SEM image and (Fig. 6). The TC values of the OD/OD-*g*-MWCNT (1:1)-5wt%, OD/OD-*g*-MWCNT (2:1)-5wt%, and OD/OD-*g*-MWCNT (4:1)-5wt% samples were higher than that of OD/MWCNT-5wt% mainly due to the better dispersion of the MWCNT within the composite structure. Again, a similar trend of reduction of the TC values were observed mainly due the increasing amount of OD in the grafted structure, but this helped to maintain the heat storage capacity of the composites very close to that of pure OD.

In Table 4, the TC results of the prepared OD/OD-*g*-MWCNT (4:1)-5wt% composite PCM is compared with those of grafted and non-grafted fatty alcohol included-CPCMs. As seen from the tabulated data, the CPCM prepared in this work had much superior thermal properties than the other similar reported PCMs^13–15^. Especially when compare with the reports of Wang *et al*.^15^, where the authors worked with OD and mixed with graphene oxide to improve its thermal properties. In comparison to pure OD, the reduction of melting enthalpy with the best performing composite sample was reported as 2.76 J/g^15^. However, in this study, the reduction of melting enthalpy was found to be 1.6 J/g, which confirms significant improvement of heat storage capacity. In case of TC, the improvement was much larger. Therefore, grafting OD on the MWCNT found to be a better option to improve its distribution within the composite structure. At the same time, it contributed towards better retention of heat storage capacity.

**Table 4:** Comparison of the TC values of grafted and non-grafted fatty alcohol included-CPCMs.

CPCM	TC value (W/mK)	TC (% increase)	Reference
Cetyl alcohol/HDPE/CF (5 wt%)	0.47	256.68	^13^
Stearyl alcohol/HDPE/EG (3 wt%)	0.67	240.69	^14^
Octadecanol/Graphene (1.5%)	0.36	44.35	^15^
Paraffin/Octanol-g-CNT (4%)	0.77	234.78	^22^
Paraffin/Tetradecyl alcohol-g-CNT (4%)	0.78	239.13	^22^
Paraffin/Stearyl alcohol-g-CNT (4%)	0.79	243.47	^22^
OD/MWCNT-5wt%	0.57 ± 0.05	256.3	*This work*
OD/OD-*g*-MWCNT(4:1)-5wt%	0.58 ± 0.04	262.5	*This work*

## Conclusions

In a significant improved approach, ODs were successfully grafted on the MWCNT in different OD to MWCNT weight ratios, and was used as conductivity filler to enhance over-all thermal properties of OD for thermal energy storage. Grafting was confirmed by the FTIR and XRD analysis and the grafted sample had better dispersion and the presence of additional OD boosted the heat storage capacities of the prepared composite PCMs as compare to the sample with pure MWCNT. No additional chemical interaction was observed in the composite PCMs and it was confirmed by same FTIR and XRD analysis. Overall, all the composite PCM samples had good thermal, chemical and cycling reliability. Champion composite PCM sample, OD/OD-*g*-MWCNT (4:1), showed a good melting and solidification enthalpy of 267.7 and -263.6 J/g respectively, which were very close to that of pure OD. Significant enhancement of thermal conductivity was achieved, which was nearly 262.5% higher than that of pure OD. This method proves to be efficient approach to improve thermal properties of the fatty alcohol and can be useful for other organic PCMs as well. Eventually a better performing PCM can be obtained for better heat storage or heat management technology.
